# Nanostructured Lipid Carriers Can Enhance Oral Absorption of Khellin, a Natural Pleiotropic Molecule

**DOI:** 10.3390/molecules26247657

**Published:** 2021-12-17

**Authors:** Giulia Vanti, Lucrezia Muti, Mario D’Ambrosio, Lucia Grifoni, Maria Camilla Bergonzi, Cristina Luceri, Anna Rita Bilia

**Affiliations:** 1 Department of Chemistry, University of Florence, Via Ugo Schiff 6, Sesto Fiorentino, 50019 Florence, Italy; giulia.vanti@unifi.it (G.V.); lucrezia.muti@stud.unifi.it (L.M.); lucia.grifoni@unifi.it (L.G.); mc.bergonzi@unifi.it (M.C.B.); 2 Department of Neurofarba, University of Florence, Viale Pieraccini 6, 50139 Florence, Italy; mario.dambrosio@unifi.it (M.D.); cristina.luceri@unifi.it (C.L.)

**Keywords:** khellin, nanostructured lipid carriers, oral administration, lyophilization, release study, gastrointestinal stability, shelf life, Caco-2 cells, cell viability, permeability

## Abstract

A novel formulation based on nanostructured lipid carriers (NLCs) was developed to increase solubility and intestinal absorption of khellin. K-NLCs were prepared with stearic acid, hempseed oil, Brij S20, and Labrafil M 1944 CS, using the emulsification-ultrasonication method. Developed nanoparticles were chemically and physically characterized by liquid chromatography, light scattering techniques, and electron microscopy. The size, about 200 nm, was optimal for oral delivery, and the polydispersity index (around 0.26), indicated high sample homogeneity. Additionally, K-NLCs showed a spherical morphology without aggregation by microscopic analysis. The encapsulation efficiency of khellin was about 55%. In vitro release studies were carried out in media with different pH to mimic physiological conditions. K-NLCs were found to be physically stable in the simulated gastric and intestinal fluids, and they preserved about 70% of khellin after 6 h incubation. K-NLCs were also successfully lyophilized testing different lyoprotectants, and obtained freeze-dried K-NLCs demonstrated good shelf life over a month. Lastly, permeability studies on Caco-2 cells were performed to predict khellin passive diffusion across the intestinal epithelium, demonstrating that nanoparticles increased khellin permeability by more than two orders of magnitude. Accordingly, developed NLCs loaded with khellin represent a versatile formulation with good biopharmaceutical properties for oral administration, possibly enhancing khellin’s bioavailability and therapeutic effects.

## 1. Introduction

Khellin, a furanochrome (4,9-dimethoxy-7-methylfuro[3,2-g]chromen-5-one), is the principal constituent of *Ammi visnaga* L. (Apiaceae), a plant widely used in folk medicine for various diseases, mainly inflammation-related illnesses [1]. In combination with ultraviolet light A (KUVA), this molecule is used to treat vitiligo as a photosensitizer [2]. The main advantage of khellin given orally at 100 mg, 2 h before treatment, is the lack of phototoxicity, making KUVA safe for use either as home treatment or treatment involving natural sunlight [3]. Khellin is also less mutagenic and produces a trivial pigmentation of normal skin when compared with psoralens’ treatment [4].

Besides the high therapeutic potential, khellin has a very insignificant solubility in water (25 mg/100 mL) and the partition coefficient between octanol and water partition (LogP) is about 3 [5], limiting the systemic absorption after oral administration. Indeed, the fraction of dose absorbed following oral suspension administration was only 38% [6], while the ideal value of logP for optimal intestinal absorption is in the range between 1.35 and 1.80 [7]. Indeed, the logP of drugs is generally used to predict their biopharmaceutical properties, including the propensity for lymphatic transport, intestinal permeability, potential pre-systemic clearance, or drug disposition. Drugs with logP in the range from 2 and 5 usually need appropriate formulations to overcome limiting biopharmaceutical properties [7].

Lipid-based formulations represent a widely used approach for poorly water-soluble drugs, which has fostered the development of several marketed drug products due to their ability to enhance drug bioavailability in vivo [8,9,10]. Accordingly, khellin is a good candidate to be loaded in lipid formulations made of different kinds of lipids (long-chain and medium-chain glycerides, waxes, and fatty acids), surfactants, and cosolvents. The choice of formulation excipients is also fundamental to properly solubilize the active ingredient and improve its pharmacokinetics and stability [11,12,13,14].

Nanostructured lipid carriers (NLCs) are among the most extensively studied lipid formulations as they can be produced on a large scale using the existing machinery at an economical cost. NLCs are made of a blend of solid and liquid lipids, which generate structural imperfections, plus surfactants, used to stabilize the particles in the dispersion medium. NLCs are reported to overcome the shortcomings of solid lipid nanoparticles due to the higher drug loading capacity, faster release rate, and low extrusion of the entrapped drug during storage [15]. NLCs’ oral administration offers numerous advantages over conventional formulations, i.e., increased drug solubility, enhanced dissolution rate and improved permeability, reduced metabolism, and, in most cases, increased bioavailability of drugs, besides the high biocompatibility of the excipients. Generally, NLCs can give a controlled release of drug from the carrier, avoiding or reducing the first-pass metabolism by lymphatic transport and providing protection to drug degradation in gut media [16,17].

In this study, K-NLCs were prepared using *Cannabis sativa* L. seed oil as a liquid lipid for its nutritional value as it is rich in omega-3 and omega-6 essential fatty acids. Brij S20, a non-ionic water-soluble surfactant, was selected as a steric stabilizer of the nanoparticles due to the hydrophilic chains, while Labrafil M 1944 CS, a non-ionic water-dispersible surfactant, was selected as a solubilizer and absorption enhancer [18] for its stability to lipase digestion [19]. Stearic acid was used as a lipid component stable at acid pH and as excipient with nutritional value as a precursor of lipid membranes. The emulsification-ultrasonication method was selected as it is inexpensive and avoids the use of toxic organic solvents [20,21]. Light scattering techniques, transmission electron microscopy, and liquid chromatography were used to determine size, polydispersity index, ζ-potential, and morphology of the nanoparticles, and recovery and encapsulation efficiency of khellin. In vitro release of khellin and physical and chemical stability of K-NLCs were carried out in solutions simulating gastric and intestinal media. K-NLCs were freeze-dried using different types and concentrations of lyoprotectants to avoid physical and chemical instability and microbial contamination during the storage. The shelf life of freeze-dried K-NLCs was evaluated over a month. Cell viability tests and permeability studies on Caco-2 cells were performed to predict khellin passive diffusion across the intestinal epithelium.

## 2. Results and Discussion

### 2.1. Preparation of Khellin-Loaded NLCs

Khellin-loaded NLCs (K-NLCs) were prepared by the emulsification-ultrasonication method. Different surfactants (Poloxamer 188, Tween 80, soy lecithin, Labrafil and Brij S20, in the range between 0.5–1%) and two solid lipids (stearic acid and Precirol in the range between 0.5 and 1%) were screened to prepare the NLCs. However, none of the tested mixtures did gave NLCs, but gelation, lump formation, aggregation, and persistent foam were observed. The only formulation able to produce NLCs was a mixture of stearic acid (0.7% *w*/*v*), hempseed oil (0.3% *w*/*v*), Labrafil (0.6% *w*/*v*), Brij S20 (0.9% *w*/*v*), and khellin (0.075% *w*/*v*).

In particular, Labrafil M 1944 CS improved the lipid phase blending with khellin, and Brij S20 had an optimal HLB to stabilize the formulation due to steric effects. The hempseed oil was used to improve khellin solubilization and give additional nutritional value to the formulation. Hence, hempseed oil, is edible and has high nutritional value as it contains omega-3 and omega-6 essential fatty acids and vitamins E, B1, B2, C, A, and PP. Hempseed oil contains 59–98% of essential fatty acids (EFAs): linoleic acid, omega-6 (46–65%); alpha-linolenic acid, omega-3 (12–28%); oleic acid (9–20%); palmitic acid (4–9%); and gamma-linolenic acid, omega-6 (1–5%). Hempseed oil is obtained by cold pressure without solvents and filtered only mechanically to obtain a commercial oil valid as a dietary supplement. During nanoparticle preparation, hempseed oil was heated to a temperature lower than its decomposition point at 124.6 °C. This temperature corresponded both to formation of hydroperoxides and decomposition of different fatty acid moieties in three sequential steps attributable to the loss of PUFA (polyunsaturated fatty acids), MUFA (monounsaturated fatty acids), and SFA (saturated fatty acids). At a temperature range of 7–125 °C, only a slight loss of moisture and volatile compounds in oils was observed. Accordingly, this vegetable oil preserves its nutraceutical properties even after its encapsulation in NLCs.

### 2.2. Optimization and Physical Characterization of K-NLCs

**Figure 1:**
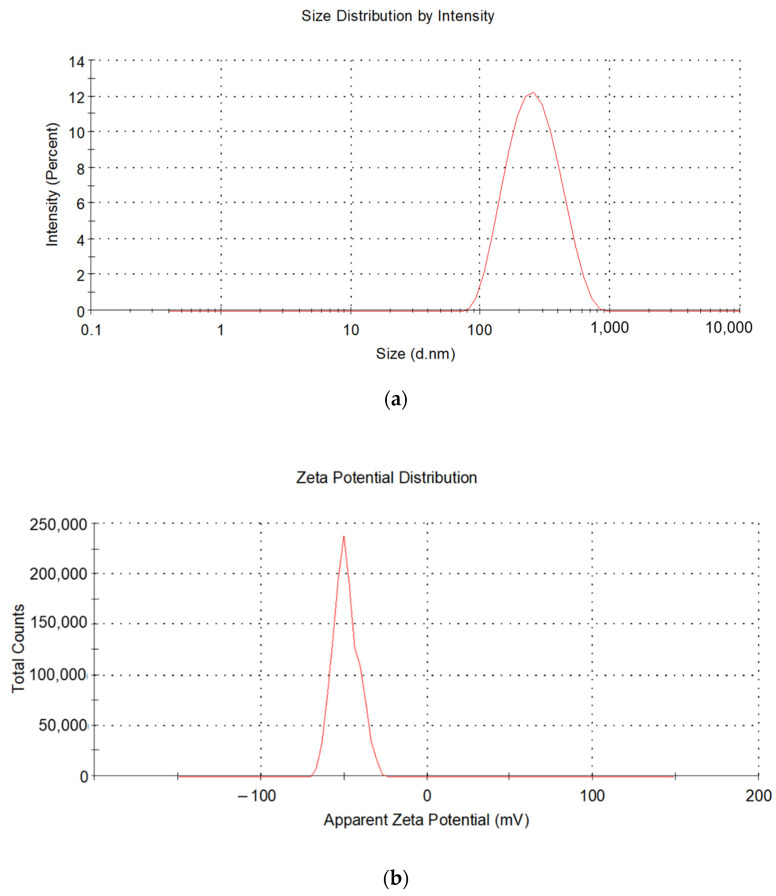
(**a**) Size and (**b**) ζ-potential distribution obtained by dynamic and electrophoretic light scattering, respectively.

Optimization of K-NLCs was carried out by changing the time, number, and temperature of ultrasonication cycles and the cooling temperature of the sample in the final step of NLC production (Table 1). These factors mainly influenced the size, polydispersity index (PdI), and repeatability of the formulation, evaluated by dynamic light scattering technique (Table 1). Notably, it was observed that a short sonication time gave smaller nanoparticles dimensions and lower PdI compared to longer ultrasonication. In addition, keeping the sample at room temperature (25 ± 2 °C) or immersing the sample in water (20 ± 2 °C) or ice to cool during and after the ultrasonication greatly affected these parameters. Finally, 2 min of ultrasonication at room temperature plus 1 min in water and cooling at room temperature were selected as the best conditions to achieve nanoparticles which were around 200 nm and a PdI around 0.2. Therefore, the average particle size is suitable for oral administration [22], and the PdI indicates a good sample homogeneity (Figure 1a). Moreover, the high ζ-potential (−50.56 ± 11.63 mV), measured by the electrophoretic light scattering technique, indicates the high physical stability of the formulation to aggregation phenomena (Figure 1b).

### 2.3. Lyophilization Process

The lyophilization process was adopted to avoid physical and chemical instability and microbial contamination during the storage [23,24]. The nanoparticle dispersion was initially freeze-dried without lyoprotectants, obtaining an increase in size and PdI. The whole process often induces sample alterations and results in inhomogeneous dispersion after water addition to the freeze-dried powder. Therefore, five types of lyoprotectants were tested (D-glucose anhydrous, D-mannitol, D-trehalose dihydrate, D-sucrose, and D-galactose) at different concentrations (2.5, 5, and 7.5% *w*/*v*). Freezing in the freezer at −24 °C was discarded as the formulation often was not wholly frozen after 3 days. By contrast, freezing with liquid nitrogen was fast, easy, and efficient.

**Figure 2:**
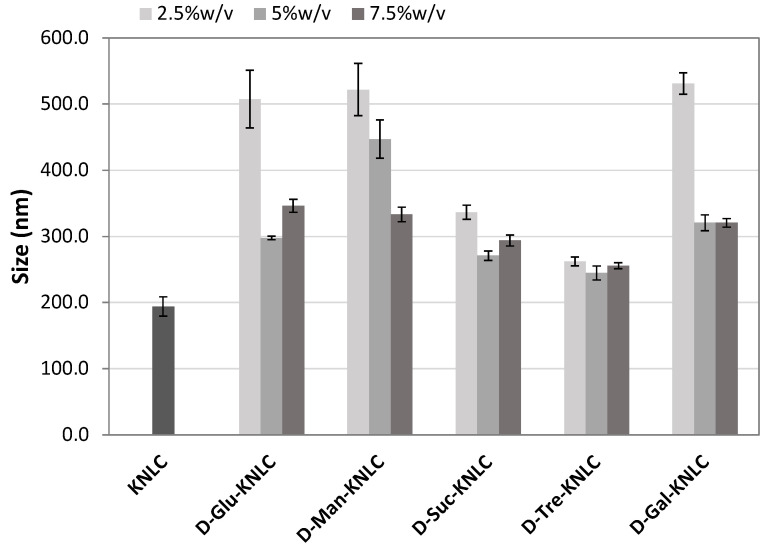
Size values of K-NLCs and K-NLCs freeze-dried using (2.5, 5, and 7.5)% *w*/*v* of different lyoprotectants (D-glucose anhydrous, D-mannitol, D-sucrose, D-trehalose dihydrate, and D-galactose). Data are shown as the mean ± standard deviation (*n* = 3).

**Figure 3:**
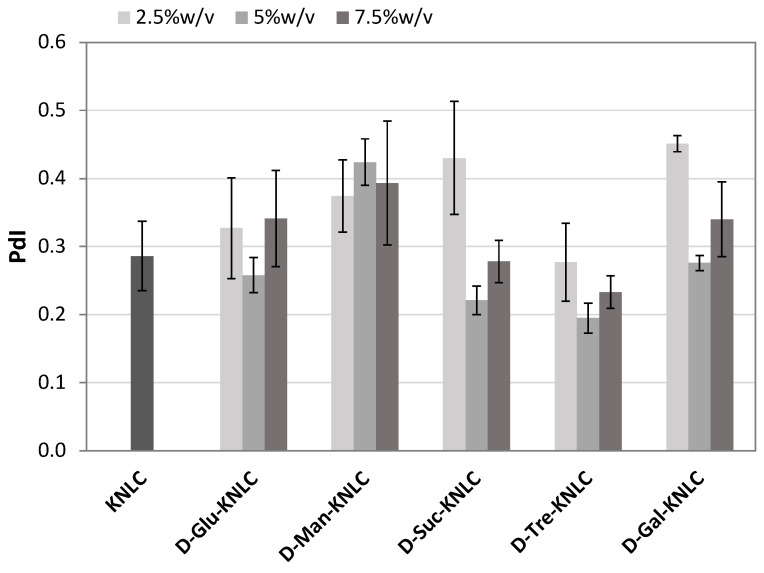
PdI values of K-NLCs and K-NLCs freeze-dried using (2.5, 5, and 7.5)% *w*/*v* of different lyoprotectants (D-glucose anhydrous, D-mannitol, D-sucrose, D-trehalose dihydrate, and D-galactose). Data are shown as the mean ± standard deviation (*n* = 3).

Size and PdI were measured as screening parameters for the selection of the lyoprotectant and its concentration (Figure 2 and Figure 3). The lowest concentration (2.5% *w*/*v*) of D-glucose anhydrous, D-mannitol, D-sucrose, and D-galactose did not enable us to preserve dimensions and PdI of the nanoparticles (Figure 2 and Figure 3). The 5% *w*/*v* concentration induced dimension increase (except for D-trehalose dihydrate) (Figure 2), but on the other hand, it decreased the PdI (except for D-mannitol) (Figure 3). However, the highest concentration (7.5% *w*/*v*) gave higher PdI values (Figure 3). The best results were obtained for 5% *w*/*v* D-trehalose dihydrate as PdI was almost unchanged after the lyophilization, while ζ-potential was increased in absolute value, confirming the high repulsion between the nanoparticles. An increase of average dimensions was observed (Table 2), but they were still compatible with the oral administration route.

**Figure 4:**
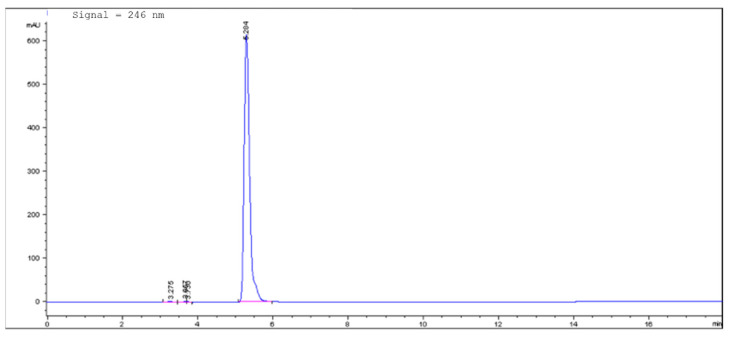
HPLC-DAD chromatogram of khellin at 246 nm.

Additionally, quantitative analyses before and after the lyophilization process, to determine recovery (R%) and encapsulation efficiency (EE%) of khellin, were carried out using an 1100 High-Performance Liquid Chromatograph (HPLC) equipped with a Diode Array Detector (DAD). Figure 4 displays the HPLC-DAD chromatogram of khellin at 246 nm. Performed analyses showed that R% and EE% of khellin remained constant during the lyophilization (Table 2). The high R% value (about 100%) before and after the lyophilization demonstrated that khellin was not degraded during nanoparticle preparation or lyophilization. Similarly, the EE% value, which indicates the percentage of khellin encapsulated in NLCs, remained unchanged after this treatment (Table 2). Therefore, overall, K-NLCs were stable to the lyophilization process with 5% *w*/*v* of D-trehalose dihydrate, one of the most widely used lyoprotectants in commercial products.

### 2.4. Morphologic Analysis by Transmission Electron Microscope

**Figure 5:**
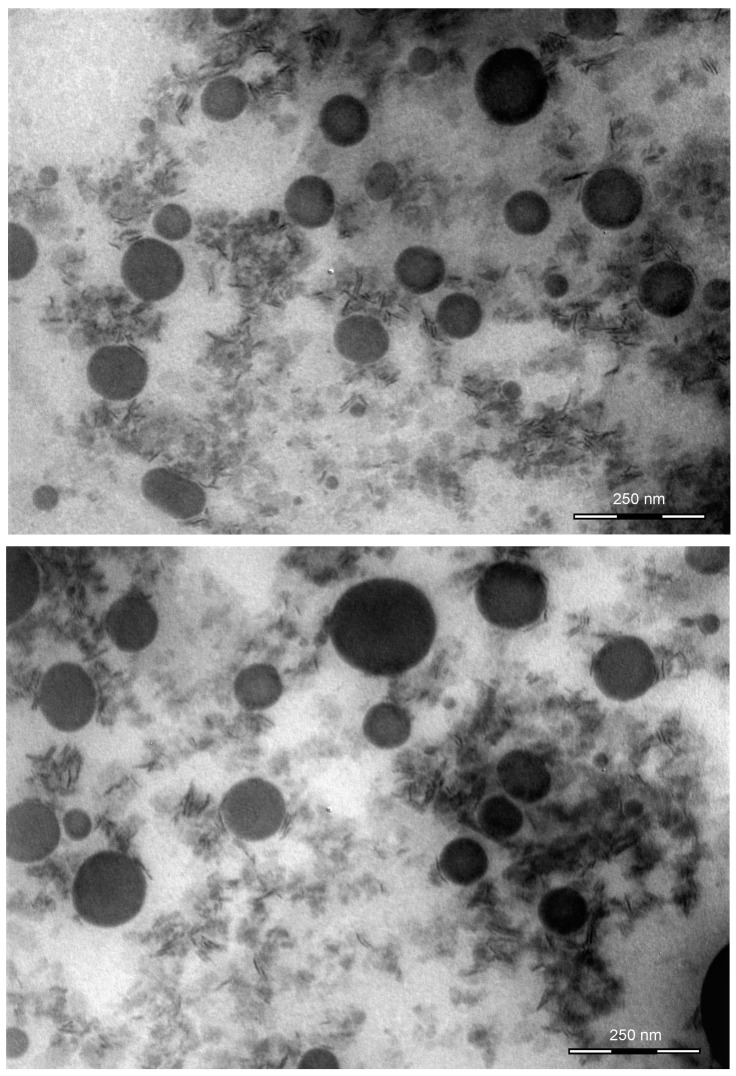
Pictures of K-NLCs obtained by transmission electron microscopy. Scale bars = 250 nm.

K-NLCs were analyzed by transmission electron microscopy. Nanoparticles had a spherical shape, smooth surface, and dimensions consistent with those detected by DLS (Figure 5). The pictures also confirmed the absence of aggregates in the formulation.

### 2.5. In Vitro Release Study of K-NLCs

**Figure 6:**
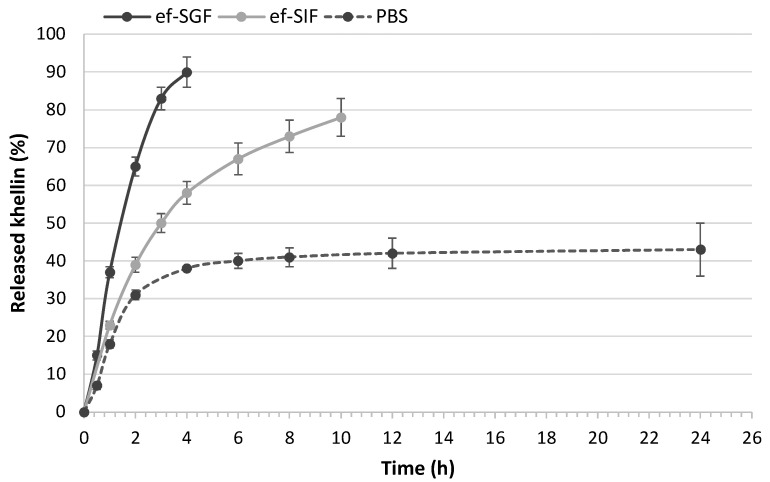
Release study of khellin from K-NLCs in different media: PBS, ef-SGF (enzyme-free simulated gastric fluid), and ef-SIF (enzyme-free simulated intestinal fluid). Data are expressed as media ± standard deviation (*n* = 3).

The in vitro release of khellin from NLCs was performed using the dialysis bag method and PBS (pH 7.4), enzyme-free simulated gastric fluid (ef-SGF, pH 2), and enzyme-free simulated intestinal fluid (ef-SIF pH 7.0) as acceptor media. A linear kinetic profile was observed for the first 2 h in all the media (Figure 6). Khellin release in PBS reached 40% after about 4 h and remained constant until 24 h (Figure 6), but longer times have not been tested as the permanence of a substance hardly exceeds four hours in the stomach [25] and six hours in the small bowel [26], where most of the absorption of nutrients, nanoparticles, and drugs take place [27]. By contrast, about 80% of khellin was released in ef-SIF after 10 h and about 90% in ef-SGF after 4 h (Figure 6). Obtained results evidenced that khellin release from developed nanoparticles was affected by the medium pH and composition.

Furthermore, these findings indicate that the freeze-dried K-NLCs can be administered orally using capsules or gastro-resistant capsules based on the need for a short or long-term release in the stomach or intestine, respectively.

### 2.6. Stability of K-NLCs in Gastric and Intestinal Simulated Fluids

The stability of K-NLCs in simulated gastric fluid (SGF) and simulated intestinal fluids (SIF) was evaluated by measuring size, PdI, and R% of khellin after different incubation times. K-NLCs were diluted 150-fold in SGF, or 10-fold in SGF and subsequently 15-fold in SIF. The total dilution 1:150 was selected to obtain more consistent data, as the same dilution was applied for the permeability tests on Caco-2 cells. Specifically, a lower dilution in SGF was carried out as the amount of water in the stomach is less than that in the intestine [28].

An increase in size is usually an index of aggregation, often caused by hydrolysis of surfactants; on the contrary, a decrease in size can be due to rapid enzymatic degradation of the system [27]. In this study, size and PdI remained constant after 2 h incubation in SGF. Size slightly increased, but not significantly, after adding SIF and incubating for 2 h more. By contrast, they increased significantly after incubating for 4 h more (Table 3). However, PdI did not increase significantly after 4 h incubation in SIF. During the experiment, any degradation due to the acidity of gastric medium or enzymatic digestion (pepsin, pancreatin, and lipase) was evidenced by sample visual inspection and by size and PdI measurement. Additionally, aggregation phenomena can be excluded as, despite nanoparticle average diameter rise, sample homogeneity was preserved, and the size increase, about 37 nm, cannot be associated with nanoparticle gathering. Therefore, K-NLCs can be considered physically stable in SGF and SIF. Surfactant characteristics are crucial for the stability of nanoparticles. Specifically, Labrafil M 1944 CS is reported to be stable at lipase digestion due to lacking classical triglyceride structure [19]. The estimated pKa of stearic acid is about 4.75, indicating that this compound will be stable at gastric pH. Furthermore, Brij S20 derives from stearic acid covalently grafted with PEG 1000 chains, which form a hydrophilic steric barrier around the NLCs, increasing their stability. The longer the ethylene oxide chains, the more the interaction with the lipases is hindered. The hindrance of the surfactant can influence the degradation rate, slowing it down but sometimes accelerating it, and can stabilize the nanoparticles physically, avoiding flocculation and coalescence phenomena [28].

Total khellin concentration, measured as R%, was also unchanged after 2 h incubation in SGF, while it significantly decreased after an additional 2 h and 4 h incubation in SIF (Table 3). This reduction can be mainly attributed to the enzymatic degradation of not encapsulated khellin as it was observed from 2 h incubation in SIF. However, at the same time, size remained stable, and PdI transiently increased (2 h), probably for a temporary arrangement of nanoparticles in the intestinal fluid, and then decreased (4 h).

Overall, K-NLCs were found to be physically stable in the simulated gastric and intestinal fluids, and they preserved about 70% of khellin after 6 h incubation. As former studies reported that the primary absorption of some kinds of NLCs occur in the duodenal region of the intestine after they pass through the stomach [25], these findings make K-NLCs a promising formulation to deliver khellin by the oral administration route.

### 2.7. Shelf Life of Freeze-Dried K-NLCs

**Figure 7:**
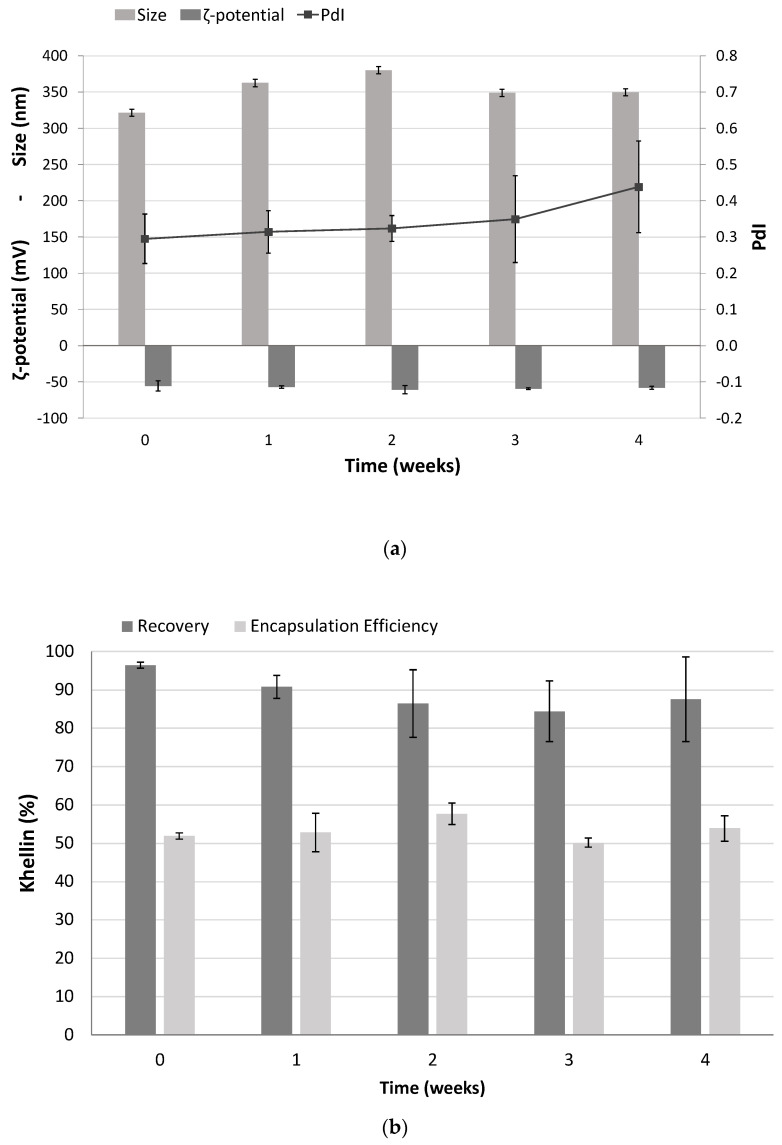
Shelf life of freeze-dried K-NLCs in terms of: (**a**) size, PdI and ζ-potential; (**b**) recovery and encapsulation efficiency of khellin. Data are shown as the mean ± standard deviation (*n* = 3).

The shelf life of freeze-dried K-NLCs was evaluated by storing the sample in a glass vacuum desiccator and analyzing size, PdI, ζ-potential, R%, and EE% weekly. After 4 weeks of storage, any aggregation or aspect change was visually observed. The average size and PdI slightly increased up to about 350 nm and 0.4, respectively, but they were still suitable for oral administration. By contrast, the ζ-potential remained constant at around −55 mV during the monitoring period (Figure 7a). R% and EE% of khellin were almost unchanged, confirming that any degradation or extrusion of khellin from the nanoparticles occurred during the storage period (Figure 7b). Overall, the freeze-dried K-NLCs demonstrated good physical and chemical stability over time.

### 2.8. Cell Viability

**Figure 8:**
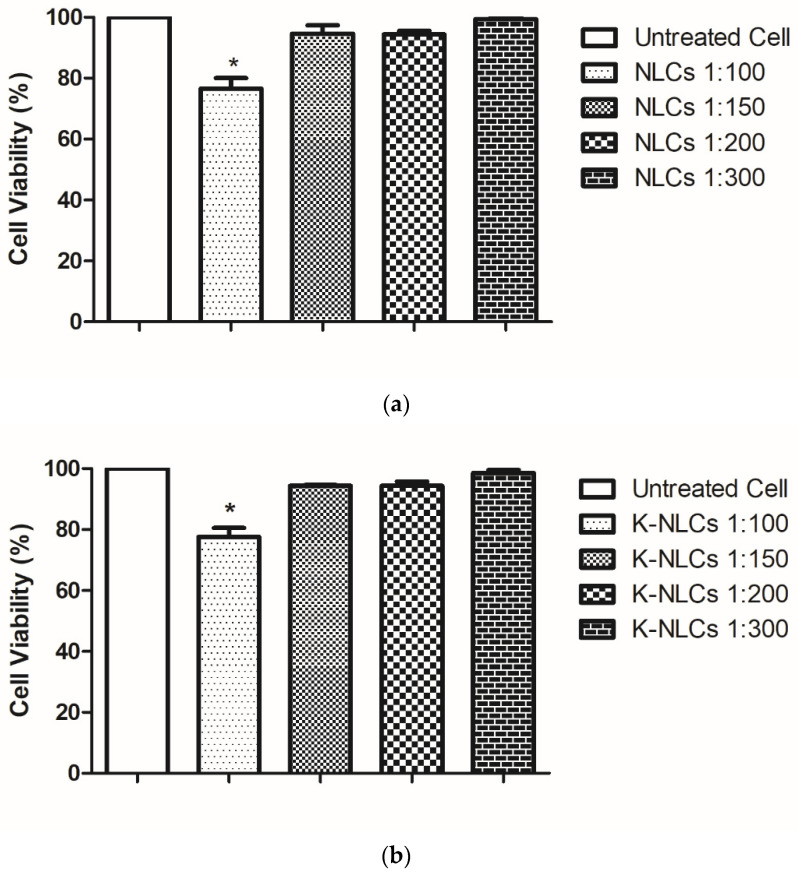
Cytotoxicity on Caco-2 cell line (% vs. untreated cells) of (**a**) NLCs and (**b**) K-NLCs at different dilutions (1:100, 1:150, 1:200, and 1:300) after 3 h of exposure. Data are shown as mean ± standard error (*n* = 3). * = *p* < 0.05.

The cytotoxicity of loaded and unloaded NLCs was first investigated by MTS assay to select the appropriate dilution for the permeability studies. When NLCs and K-NLCs were diluted 1:150, cell viability was not significantly reduced compared to the control group of untreated cells. After 3 h exposure, only 5% decrease of cell viability was observed (Figure 8). Therefore, 1:150 was selected as dilution ratio for permeability studies. Additionally, this value may reproduce in vivo conditions as oral formulations undergo a dilution from 1:200 to 1:1000 with biological fluids after oral administration [29,30].

### 2.9. Permeability Study on Caco-2 Cells

Caco-2 cells are considered the most widely used predictive model to estimate passive diffusion, active transport processes, paracellular permeability, and active efflux across the intestinal epithelium [31]. In particular, Caco-2 cells are rich in mucin content as previously demonstrated [32]. For this reason, Caco-2 cell line also represent a suitable model to preliminarily predict the effects of mucus layer of the intestinal epithelium during permeation. In the present study, the permeability of khellin by passive diffusion was evaluated by comparing unformulated khellin (PBS solution) and formulated khellin (K-NLCs) at the same concentration, after 2 h incubation in GIF plus 2 h in SIF. The apparent permeability (*P_app_*), measured after 2 h incubation with Caco-2 cells, was (1.15 ± 0.06) × 10^−8^ cm/s for khellin solution and (4.96 ± 1.25) × 10^−6^ cm/s for K-NLCs. This enhancement could be ascribed to the formulation surfactants [33,34]. Specifically, Labrafil M 1944 CS works as a penetration enhancer and solubilizing agent of poorly water-soluble active ingredients [18], while Brij S20 has been reported to present inhibitory effects on Pgp function [29,35]. Therefore, this study demonstrated that lipid nanoparticles increased khellin permeability across the Caco-2 cell monolayer and mucus layer produced by cells by more than two orders of magnitude, possibly enhancing therapeutic effects. Furthermore, according to the literature, PEG-coating or PEGylation strategies enhance nanoparticle diffusion in the mucus layer. Developed K-NLCs were formulated with two PEGylated surfactants, Labrafil M 1944 CS and Brij S20, and these surfactants can contribute to enhance mucus-penetrating nanoparticles properties optimizing the drug delivery to the gastrointestinal tract and mucosal surface [36].

## 3. Materials

### 3.1. Chemicals

Khellin (95%) and *Cannabis sativa* L. seed oil were purchased from Galeno Srl (Prato, Italy). Stearic acid, Brij S20, calcium chloride, sodium chloride, D-mannitol, D-trehalose dihydrate, sodium hydroxide, lipase from porcine pancreas, pancreatin from porcine pancreas, pepsin from porcine gastric mucosa, sucrose, bile salts, phosphate-buffered saline, and all the solvents (HPLC grade acetonitrile, HPLC grade formic acid, hydrochloric acid, 96% ethanol) were bought from Sigma Aldrich (Milano, Italy). Phosphotungstic acid (PTA) was from (Hatfield, PA, USA), while D-glucose anhydrous and D-galactose were purchased from Fluka^®^ Analytical (Munich, Germany). Labrafil M 1944 CS was generously gifted by Gattefossè (Saint Priest, Germany). Ultrapure water was produced using a synergy UV Simplicity water purification system provided by Merck Life Science Srl (Milano, Italy).

### 3.2. Cell Culture

The colorectal adenocarcinoma cell line (Caco-2) was purchased from American Tissue Type Culture Collection (Manassas, VA, USA) and cultured in Dulbecco’s modified Eagle’s medium (DMEM; Thermo Fisher Scientific, Rodano, Milan, Italy), with 20% fetal bovine serum (FBS; Thermo Fisher Scientific, Rodano, Milan, Italy), 100 U/mL penicillin-streptomycin and 1% L-glutamine (Thermo Fisher Scientific, Rodano, Milan, Italy), at 37 °C in a humidified atmosphere containing 5% CO_2_. Upon reaching 80% confluence, cells were sub-cultured weekly at a split ratio of 1:3 by trypsinization.

## 4. Methods

### 4.1. Quantitative Analysis by High Performance Liquid Chromatography

Quantitative analyses of khellin were carried out using an 1100 High-Performance Liquid Chromatograph (HPLC) equipped with a Diode Array Detector (DAD) (Agilent Technologies Italia Spa; Rome, Italy), and an Eclipse XDB-C18 column with an internal diameter of 3.5 μm and (150 × 4.6) mm dimensions [37]. All the analyses were performed using a gradient analytical method with a flow rate of 0.4 mL/min and a mobile phase composed of (A) formic acid/water (pH 3.2), (B) acetonitrile. The multi-step linear solvent gradient used was: 0–2 min: 50% B; 2–13 min 50–80% B; 13–15 min 80–50%; 15–18 min 50%; and post time was 5 min. The temperature was set at 25 °C, and the UV-visible spectra were recorded in the range of 200–600 nm. All chromatograms were acquired at 246 nm [38]. A linear relationship was obtained in the investigated concentration range of 10–0.05 µg (R^2^ = 0.9999).

### 4.2. Preparation of Khellin-Loaded NLCs

Khellin-loaded NLCs (K-NLCs) were prepared using the emulsification-ultrasonication method [20,21]. The aqueous phase was prepared by solubilizing 180 mg of Brij S20 (0.9% *w*/*v*) in 20 mL of PBS, while the lipid phase was composed of: 7% *w*/*v* of stearic acid, selected as solid lipid, 3% *w*/*v* of *Cannabis sativa* L. seed oil, selected as liquid lipid, and 6% *w*/*v* of Labrafil M 1944 CS (HLB ≈ 9), chosen as a lipophilic surfactant (HLB required for stearic acid and hemp oil: 15 and 7, respectively). Percentages of lipid components are related to the volume of the aqueous phase. Khellin (0.75% *w*/*v* of the aqueous medium) was added and dissolved in the lipid phase. Aqueous and oil mixtures were, separately but simultaneously, placed in a water bath at 85 ± 2 °C, under magnetic stirring at 250 rpm, for 20 min, to allow the components to melt or dissolve and homogeneously mix. The water bath temperature was carefully controlled by a thermometric probe. Then, the two hot solutions were quickly mixed, adding the aqueous phase all in once to the oil phase, and emulsified using the T25 Ultra-Turrax (IKA; Khonigswinter, Germany) at 5000 rpm speed for 6 min. The resulting microemulsion was immediately optimized using the ultrasonic homogenizer Sonopuls HD 2200 (Bandelin Electronic GmbH; Berlin, Germany) coupled to the KE-76 probe. The ultrasonication process was carried out with 1/2 s on and 1/2 s off cycles at 10% power. The first 2 min cycle was performed at room temperature (25 ± 2 °C), while the second 1 min cycle was carried out keeping the sample in a water bath at 20 ± 2 °C.

### 4.3. Lyophilization Process

Prepared K-NLCs were freeze-dried using different lyoprotectants [23,24]. D-glucose anhydrous, D-mannitol, D-trehalose dihydrate, D-sucrose, and D-galactose were tested at the different concentrations: (2.5, 5, and 7.5)% *w*/*v* lyoprotectants were solubilized in the K-NLCs dispersion, and the mixture was kept under magnetic stirring at 300 rpm for 20 min. Obtained samples were divided into about 4 mL aliquots and were frozen by slow freezing in the freezer (−24 °C) for three days or by fast freezing using liquid nitrogen. Subsequently, the samples were freeze-dried for at least one day through the Leybold-Heraeus freeze-drier Lyovac GT (Leybold; Turin, Italy). After that, samples were reconstituted with the original volume of ultrapure water and were stirred at 300 rpm for 20 min to ensure the complete redispersion of the powder.

### 4.4. Characterization by Light Scattering Techniques

K-NLCs were analyzed by dynamic and electrophoretic light scattering (DLS and ELS) techniques, using the Zetasizer Nanoseries ZS90, equipped with a 4 mW He-Ne laser, operating at 632.8 nm, and an APD detector (Malvern Panalytical Ltd.; Worcestershire, UK). Time correlation functions were analyzed using the Zetasizer software version 7.02, provided by Malvern, and all data were analyzed by the Cumulants method [39,40]. Average hydrodynamic diameter (size, nm), polydispersity index (PdI, dimensionless measurement), and ζ-potential (mV) were measured during formulation development, stability studies, and after the freeze-drying process. All measurements were carried out at 25 °C using 10 × 10 × 45 mm polystyrol/polystyrene cuvettes and a scattering angle of 90 °C. K-NLCs were diluted 100-fold in ultrapure water before each analysis, while freeze-dried K-NLCs were first dispersed in ultrapure water at the initial concentration of nanoparticle dispersion and then diluted 100-fold for the analysis.

### 4.5. Characterization by Transmission Electron Microscope

Morphologic analysis of K-NLCs was carried out by transmission electron microscopy (TEM) on a TEM CM12 PHILIPS equipped with an OLYMPUS Megaview G2 camera at an accelerating voltage of 80 keV [41]. For the analyses, freeze-dried K-NLCs were dispersed in ultrapure water at the initial concentration and obtained nanoparticle dispersion was placed on a carbon film-covered copper grid. Subsequently, the excess of the sample was blotted from the grid with filter paper to obtain a thin film stained with a phosphotungstic acid solution (1% *w*/*v*) in distilled water. The analysis of the samples was performed 3 min after the staining.

### 4.6. Determination of Khellin Encapsulation Efficiency and Recovery

Encapsulation efficiency (EE%) of khellin in the NLCs was determined by the direct method of dialysis bag. Specifically, for the purification step, 2 mL of K-NLCs were loaded into a Spectra/Por^®^ dialysis tubing of regenerated cellulose with molecular weight cut-off of 3.5 KDa (Repligen Europe B.V.; Breda, The Netherlands) and were dialyzed against 1 L of ultrapure water at room temperature (25 °C) in stirring conditions (100 rpm) for 1 h [42,43]. Subsequently, 100 µL of purified K-NLCs were treated with 900 µL of ethanol, vortexed few seconds and heated at 65 ± 2 °C in the ultrasonic bath to break the nanoparticle structure and dissolve the drug. Obtained samples were centrifuged at 14,000 rpm for 10 min to remove suspended undissolved residues; then, the supernatants were analyzed by HPLC-DAD. The encapsulation efficiency was calculated as the percentage of the concentration of entrapped khellin (determined after the purification step) divided by the initial drug concentration (determined by weighting).

Recovery (R%) was determined with the same procedure without the purification step. Therefore, it was calculated as the percentage of the total khellin concentration divided by the initial drug concentration.

### 4.7. In Vitro Release Study

In vitro release of khellin from nanoparticles was studied using three different media: PBS (pH 7.4), enzyme-free simulated gastric fluid (ef-SGF; pH 2), and enzyme-free simulated intestinal fluid (ef-SIF; pH 7) [44]. For each release assay, 2 mL of K-NLCs were put in a Spectra/Por^®^ dialysis tubing of regenerated cellulose with molecular weight cut-off of 3.5 KDa (Repligen Europe BV; Breda, The Netherlands) and selective permeability. The dialysis tubing was kept in stirring conditions (150 rpm) in 200 mL of the medium at 37 ± 0.5 °C. At predetermined time intervals, 500 µL of medium were withdrawn in duplicate. Precisely, 30–60 min for all media and successively every 2 h until 24 h for release in PBS, every hour until 4 h for release in ef-SGF and every 2 h until 10 h for release in ef-SIF. All collected samples were analyzed by HPLC-DAD and immediately replaced with the respective fresh medium.

### 4.8. Stability Studies in Gastro-Intestinal Simulated Fluids

The stability of K-NLCs, in terms of khellin concentration (R%), size, and PdI of nanoparticles, was evaluated in simulated gastric fluid (SGF) and simulated intestinal fluid (SIF) [29]. The composition of SGF was: 3.2 g of pepsin, 2 g of NaCl, and 7 mL HCl dissolved in 1 L of water (pH adjusted to 2.0 using 1 M NaOH). A 100 μL aliquot of K-NLCs was diluted in 900 μL SGF (dilution factor 1:10) and placed in the PST-60H-4 Plate Thermo-Shaker (Biosan; Riga; Latvia) at 37 °C under continuous stirring at 300 rpm for 2 h. After digestion in SGF, the sample was diluted with dilution factor 1:15 in SIF (pH 7.0), constituted of 0.4 mg/mL lipase, 0.15 mg/mL bile salts, 0.5 mg/mL pancreatin, and 750 mM CaCl_2_. This sample was incubated further 4 h at 37 °C under magnetic stirring at 300 rpm. At the end of the incubation times (2–4 h), the sample was diluted 1:15 in ultrapure water (2 h) or analyzed as such (4 h) to measure size and PdI, while it was diluted 1:10 in EtOH for R% determination, as described in paragraph, in both cases. The total dilution of K-NLCs in SGF and SIF was 1:150 to simulate the dilution performed during cell permeation studies and the in vivo dilution after oral administration.

### 4.9. Shelf Life of Freeze-Dried K-NLCs

Freeze-dried K-NLCs were stored in a glass vacuum desiccator, and their chemical and physical stability was evaluated for one month. At weekly intervals, an exact amount of freeze-dried K-NLCs was dispersed in an exact volume of ultrapure water, to obtain the initial nanoparticle dispersion, and the sample was kept under stirring at 300 rpm for 20 min until homogeneity. Then, size, PdI, and ζ-potential were determined for physical stability, while khellin R% was evaluated for chemical stability.

### 4.10. MTS Assay

Caco-2 cells were seeded in 96-well cell culture plates, 5 × 10^3^ cells/well and allowed to grow for 24 h under the conditions detailed above. Cells were then exposed to NLCs (unloaded) or K-NLCs (loaded with khellin) at different dilutions, for 2 h. The MTS assay was performed as previously reported [29]. Cell viability was expressed as percentage of values obtained from untreated cells (controls) and treaded cells.

### 4.11. Permeability Study on Caco-2 Cells

For permeability studies, cells were seeded at 2 × 10^5^ cells/well into 12-well PET transwell plates (1.13 cm^2^ growth surface area and pore size 0.4 µm, Greiner Bio-One, Milan, Italy). Culture medium (DMEM) was added to apical (AP) (0.5 mL) and basolateral (BL) (1.5 mL) side and replaced every three days for the first week and next, daily. Cells were let to differentiate for 18–21 days.

Lucifer Yellow (LY) permeability test was applied to evaluate the integrity of the cellular barrier and the permeability study was performed as previously described [29]. Briefly, before the transport study, DMEM was replaced with preheated (37 °C) transport HBSS medium, supplemented with 25 mM HEPES (pH 7.4). After that, the cell monolayer was equilibrated for 30 min at 37 °C; then, Caco-2 cells were exposed for 2 h to K-NLCs or PBS solution of free khellin at a final dilution of 1:150, previously incubated 2 h in GIF plus 2 h in SIF. At the end of the experiment, BL and AP samples and cellular lysates were taken and analyzed by HPLC-DAD. The apparent permeability (*P_app_*, cm/s) was calculated with the following equation [29]:

(1) $$P_{app}=\frac{V_{A}}{\left(A\times C_{D}\right)\times \left(\frac{\Delta C_{A}}{\Delta t}\right)},$$

where *V_A_* is the acceptor (basal) volume (mL), *A* is the surface area (cm^2^), *C_D0_* is the concentration in the donor (apical) chamber at the start of the experiment, and Δ*C_A_*/Δ*t* is the change of drug concentration in the acceptor (basal) compartment over time (s).

### 4.12. Statistical Analysis

The experiments were performed at least in triplicate. The results were expressed as the mean ± standard deviation and analyzed by Kruskal–Wallis test and Dunn’s Multiple Comparison Test. All analyses were carried out using GraphPad Prism 7.0 (GraphPad Software, San Diego, CA, USA). A *p* value of 0.05 was considered significant.

## 5. Conclusions

This study aimed to develop and optimize NLCs loaded with khellin to improve khellin solubility, intestinal absorption, and, consequently, oral bioavailability. Food-grade excipients (GRAS, generally recognized as safe) have been chosen to avoid any toxicity deriving from the formulation. Moreover, *Cannabis sativa* L. seed oil, extracted with cold pressure method and rich in essential fatty acids, was used as a liquid lipid for its nutritional value. Stearic acid was selected as stable lipid at acid pH and for its nutritional function as a precursor of membrane lipids, while Brij S20 was used for the optimal HLB and molecular structure to stabilize the formulation and its effect on the intestinal epithelium to improve khellin permeability. Labrafil M 1944 CS was chosen for its technological properties as a solubilizing agent and absorption enhancer.

Developed K-NLCs increased khellin solubility by three-fold compared to water solubility and showed average dimensions, around 200 nm, suitable for oral delivery. In vitro release studies, carried out in solutions simulating gastric and intestinal media, indicated a pH-dependent khellin release, useful information to predict the in vivo release based on the final dosage form. K-NLCs were physically stable in the simulated gastric and intestinal fluids, and they preserved khellin (about 70%) after 6 h incubation. K-NLCs were also successfully freeze-dried, using 5% D-trehalose dihydrate, to avoid physical and chemical instability and microbial contamination during the storage. Accordingly, the stability study of freeze-dried nanoparticles demonstrated good shelf life over a month in terms of size, PdI, ζ-potential, R%, and EE%. A lyophilized drug also has many advantages and versatility in large-scale production as it can be easily stored, transported, and used to prepare other dosage forms such as tablets, capsules, pellets, granules, powders, and syrups. Permeability studies on Caco-2 cells demonstrated that nanoparticles increase khellin passage across cell monolayer by more than three orders of magnitude.

Accordingly, developed K-NLCs represent a versatile formulation that possibly enhances in vivo khellin absorption over the intestinal epithelium, improving bioavailability and therapeutic effects in diseases such as vitiligo. These promising results encourage further research to apply K-NLCs in pharmaceutical preparations such as capsules, tablets, granules, or as components of medical devices for khellin oral delivery.

## Figures and Tables

**Table 1:** Optimization of the emulsification-ultrasonication method and characterization by dynamic light scattering. Data are shown as the mean ± standard deviation (*n* = 3).

Optimization			Characterization	
1°Sonication	2°Sonication	Cooling	Size (nm)	PdI
2 min (ice)	1 min (*)	r.t.	545 ± 21	0.363 ± 0.082
2 min (r.t.)	1 min (*)	r.t.	216 ± 27	0.258 ± 0.024
2 min (r.t.)	1 min (*) + 2 min (r.t.)	r.t.	336 ± 50	0.482 ± 0.091
3 min (r.t.)	/	ice	582 ± 33	0.546 ± 0.081
3 min (r.t.)	/	ice + water *	477 ± 53	0.591 ± 0.076
5 min (water *)	/	r.t.	692 ± 64	0.392 ± 0.021
5 min (water *)	/	r.t.	251 ± 16	0.452 ± 0.025
5 min (water *)	5 min (r.t.)	r.t.	614 ± 9	0.267 ± 0.021
10 min (water *)	/	r.t.	803 ± 81	0.332 ± 0.056

r.t. = room temperature (25 ± 2 °C); * 20 ± 2 °C.

**Table 2:** Physical (size, PdI, and ζ-potential) and chemical (R% and EE%) characterization of K-NLCs and K-NLCs freeze-dried with 5% *w*/*v* D-trehalose dihydrate. Results are shown as the mean ± standard deviation (*n* = 3).

	K-NLCs	Freeze-Dried K-NLCs
Size (nm)	216.4 ± 26.8	313.0 ± 26.5
PdI	0.258 ± 0.024	0.264 ± 0.053
ζ-potential (mV)	−48.5 ± 5.6	−54.5 ± 5.85
Khellin R%	103.06 ± 6.24	96.42 ± 0.79
Khellin EE%	54.77 ± 9.12	51.94 ± 0.81

**Table 3:** Physical and chemical stability of K-NLCs, in terms of size, PdI, and recovery of khellin, respectively, in simulated gastric fluid (SGF) and simulated intestinal fluid (SIF) for different incubation times. Data are shown as the mean ± standard deviation (*n* = 6).

Incubation Time (h)	Size (nm)	PdI	Recovery (%)
0	249.7 ± 9.1	0.200 ± 0.020	97.70 ± 2.77
2 (SGF)	254.8 ± 3.2 ^ns^	0.213 ± 0.023 ^ns^	96.59 ± 3.33 ^ns^
2 (SGF) + 2 (SIF)	262.5 ± 6.8 ^ns^	0.310 ± 0.053 **	72.66 ± 8.19 *
2 (SGF) + 4 (SIF)	286.8 ± 5.5 ***	0.267 ± 0.028 ^ns^	69.83 ± 8.18 *

ns = not significant, * = *p* < 0.05, ** = *p* < 0.01, and *** = *p* < 0.001 versus the Incubation Time 0.

## Data Availability

Not applicable.

## Sample Availability

Samples of the pure constituents and formulations are available from the authors.
